# The severity of nutrition and pneumonia predicts survival in patients with aspiration pneumonia: A retrospective observational study

**DOI:** 10.1111/crj.13521

**Published:** 2022-07-05

**Authors:** Yorihide Yanagita, Shinichi Arizono, Yuichi Tawara, Masaki Oomagari, Hikaru Machiguchi, Koshi Yokomura, Norimasa Katagiri, Yuki Iida

**Affiliations:** ^1^ Department of Physical Therapy, School of Health Science Toyohashi Sozo University Toyohashi City Aichi Prefecture Japan; ^2^ Department of Rehabilitation Seirei Mikatahara General Hospital Hamamatsu City Shizuoka Prefecture Japan; ^3^ Department of Physical Therapy, School of Rehabilitation Science Seirei Christopher University Hamamatsu City Shizuoka Prefecture Japan; ^4^ Department of Respiratory Medicine Seirei Mikatahara General Hospital Hamamatsu City Shizuoka Prefecture Japan; ^5^ Department of Rehabilitation Medicine Seirei Mikatahara General Hospital Hamamatsu City Shizuoka Prefecture Japan

**Keywords:** aspiration pneumonia, mortality, nutritional status, severity of illness index

## Abstract

**Introduction:**

Aspiration pneumonia is a common problem among older adults; it has a high mortality rate and the prevalence is increasing. Reports on the risk factors for mortality in patients with aspiration pneumonia are limited. This study aimed to evaluate the risk factors for 90‐day survival in patients with aspiration pneumonia.

**Methods:**

This retrospective observational study was conducted at Seirei Mikatahara General Hospital between 1 April 2015 and 31 March 2016. Patients with aspiration pneumonia who had dysphagia or aspiration confirmed by modified water swallow test or VideoEndoscopic examination of swallowing were included. The primary endpoint was 90‐day survival. We performed univariate and multivariate logistic regression analyses with survival and non‐survival at 90 days as the independent variables.

**Results:**

A total of 276 patients were recruited for this study. The A‐DROP score (odds ratio [OR] = 2.440; 95% confidence interval [CI], 1.400–4.270; *p* < 0.01), Geriatric Nutritional Risk Index score (OR = 0.383; 95% CI, 0.178–0.824; *p* < 0.05) and sex (OR = 0.365; 95% CI, 0.153–0.869; *p* < 0.05) were independent early predictors of mortality.

**Conclusion:**

The results suggest that nutritional status and the severity of pneumonia are important factors that predict life expectancy in patients with aspiration pneumonia.

## Graphical abstract

We performed univariate and multivariate logistic regression analyses with survival and non‐survival at 90 days as the independent variables. A total of 276 patients with aspiration pneumonia were recruited for this study. The A‐DROP score, GNRI and sex were independent early predictors of mortality.

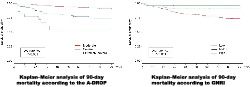

## 1 INTRODUCTION

Aspiration pneumonia is a common problem among older people; it has a high mortality rate and an increasing prevalence. The incidence of aspiration pneumonia is difficult to determine because there are few diagnostic markers for aspiration and most studies do not distinguish between aspiration pneumonia and aspiration pneumonitis.^1^ The majority of cases of aspiration pneumonia in the elderly are community‐acquired pneumonia (CAP) and nursing‐ and healthcare‐associated pneumonia (NHCAP). More than 60% of elderly patients hospitalised with CAP are considered to have aspiration pneumonia, and almost all hospitalised patients with NHCAP are considered to have aspiration pneumonia.^2^ In fact, when hospitalised cases in Japan were classified into aspiration pneumonia and non‐aspiration pneumonia according to the aetiology, 80.1% of pneumonia patients over the age of 70 were found to have aspiration pneumonia, and the proportion of aspiration pneumonia increased with age.^2^ Consequently, aspiration pneumonia is a dominant form of CAP and healthcare‐associated pneumonia (HCAP) and a leading cause of death in an ageing society. According to a previous systematic review, aspiration pneumonia was associated with significantly increased in‐hospital and 30‐day mortality compared with non‐aspiration pneumonia.^3^ A study of 47 hospitalised patients reported that the mortality rate was 90% if two or more lobes of the lung were involved and 41% if only one lobe was affected.^4^

Risk factors for the development of aspiration pneumonia include advanced age, hypoalbuminemia, chronic obstructive pulmonary disease, chronic heart failure, multiple chronic diseases, dementia, mental confusion and the number of medications.^5^ However, reports on the risk factors of mortality for aspiration pneumonia are limited. This is because there is no gold standard for the definition of aspiration pneumonia, and it is difficult to distinguish between aspiration pneumonia and typical pneumonias of CAP, NHCAP and HCAP. The prognosis of aspiration pneumonia may be related to the severity of pneumonia and respiratory failure and the nutritional status resulting from feeding disorders. This study aimed to evaluate the risk factors for 90‐day survival in patients with aspiration pneumonia. The 90‐day survival has been defined in several earlier studies as late survival. Therefore, we chose to evaluate 90‐day survival, because we wanted to confirm the longer term effects of the risk factors.

## 2 METHODS

### 2.1 Study population

This retrospective observational study was registered with the University Hospital Medical Information Network Clinical Trial Registry (UMIN 000046923).

Consecutive patients who were admitted to Seirei Mikatahara General Hospital (Shizuoka, Japan) with a diagnosis of aspiration pneumonia (International Statistical Classification of Diseases, 10th Revision, code J69) between 1 April 2015 and 31 March 2016 were enrolled. The diagnosis of aspiration pneumonia was established in cases of dysphagia and aspiration according to the Japanese guidelines,^6^ performed by three or more physicians at a conference. That the three or more physicians reviewed the cases sitting together in conference, not separately. Dysphagia and aspiration were examined using modified water swallow test or VideoEndoscopic examination of swallowing. The exclusion criteria were the disagreement with non‐purpose use of medical records, complete analysis data and bedridden level before admission.

### 2.2 Measurements

The primary endpoint was 90‐day survival, and several clinical variables were compared between the survival and non‐survival groups. A total of 28 items were surveyed. We adopted the information at the time of admission for basic subject characteristics and disease information and the information during hospitalisation for treatment details and progress. These assessments are routinely performed on patients admitted to Seirei Mikatahara General Hospital. C‐reactive protein (CRP) and white blood cell (WBC) levels were evaluated as inflammatory biomarkers.

In terms of activity of daily living (ADL), ‘freestanding gait’ was defined as the ability to walk both indoors and outdoors; ‘assistance gait’ was defined as the ability to walk with the use of an assistive device; ‘wheelchair’ was defined as the ability to stand and ride in a wheelchair; and ‘bedridden’ was defined as the inability to sit in a wheelchair on a daily basis. Performance status was rated from 0 to 4 using the Eastern Cooperative Oncology Group Performance Status Scale.^7^ The Nishimura Geriatric Rating Scale for Mental Status (NM scale)^8^ was used to classify dementia. The NM scale is a 12‐item rating scale used to observe the behaviour of patients with dementia in their daily lives from multiple perspectives. It does not require communication and can be used to assess the severity of the disease at five levels. The A‐DROP was used to classify the severity of pneumonia. The A‐DROP score, consisting of age ≥ 70 years in men or ≥75 years in women, blood urea nitrogen (BUN) ≥ 21 mg/dl or dehydration, oxyhaemoglobin saturation measured by pulse oximetry ≤ 90% or partial oxygen pressure in the arterial blood ≤ 60 mmHg, confusion and systolic blood pressure ≤ 90 mmHg, is a modified version of the CURB‐65 score proposed by the Japanese Respiratory Society in 2006.^9^ Its predictive power is similar to that of the CURB‐65 and Pneumonia Severity Index (PSI).^10^, ^11^

Survival curves for 90‐day mortality were calculated for the risk factors A‐DROP, GNRI and sex obtained by multivariate regression analysis. To determine the survival curve using the Geriatric Nutritional Risk Index (GNRI), the patients were classified into three groups: low GNRI (GNRI < 82) with severe nutritional risk, middle GNRI (82 ≤ GNRI < 92) with moderate nutritional risk and high GNRI (92 ≤ GNRI) with low or no nutritional risk, according to a previous report.^12^ The GNRI was calculated from the serum albumin and body mass index (BMI) obtained on hospital admission:

$$\text{GNRI}=14.89\times \text{serum albumin}\;\left(g/\mathrm{dl}\right)+41.7\times \mathrm{per}\;\text{cent body weight}/\left({\left[\text{height}\right]}^{2}\;\left[m^{2}\right]\times 22\right)=14.89\times \text{serum albumin}\;\left(g/\mathrm{dl}\right)+41.7\times \mathrm{BMI}\;\left(\mathrm{kg}/m^{2}\right)/22.$$

### 2.3 Statistical analyses

Differences in continuous variables were compared using the *t*‐test for normally distributed variables and the Mann–Whitney *U*‐test for non‐normally distributed variables. Categorical variables were compared using the Chi‐squared test.

We performed univariate and multivariate logistic regression analyses with survival and non‐survival at 90 days as the independent variables. Among all items, univariate logistic analysis was performed, except for outcome and ADL at discharge.

Multivariate logistic regression analysis was performed by adding age to the seven items that showed significant differences in the univariate regression analysis and using the six items, except BUN and serum albumin, which had high variance inflation factor values, as dependent variables among the combinations suspected of showing multicollinearity. Multivariate logistic regression analysis was performed using the forced imputation method.

The survival curve for 90‐day mortality after hospital admission was estimated using the Kaplan–Meier method and compared using the log‐rank test.

IBM SPSS Statistics for Windows Version 24.0 (IBM Japan, Ltd., Tokyo, Japan) was used for the analyses. The significance level for multivariate analysis was set at 5%, and 1% for other analyses.

## 3 RESULTS

### 3.1 Patient characteristics

**FIGURE 1:**
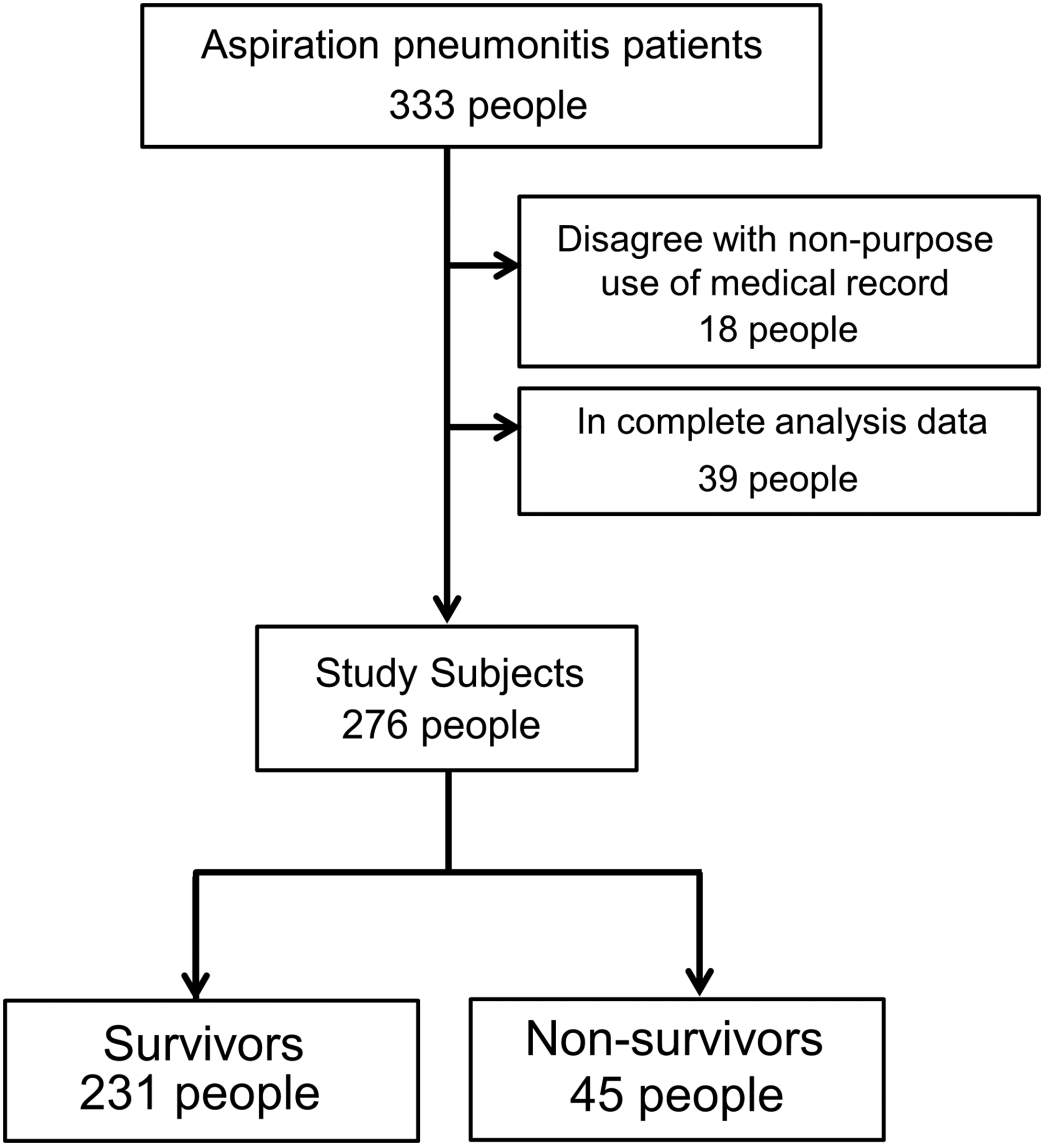
Patient selection

In total, 333 consecutive patients with aspiration pneumonitis were screened for eligibility, of whom 276 (82.9%) were recruited for the study (Figure 1). The mean age of the patients was 80.8 ± 12.9 years, and 181 (65.6%) were men.

### 3.2 Comparison between the survival and non‐survival groups

There were 231 survivors and 45 non‐survivors (Table 1). There were significant differences in 11 items in the comparison between the two groups. There was no significant difference in age between the two groups. There was also no difference in the length of hospitalisation or the history of hospitalisation within a year. However, the non‐survival group had a higher disease severity and percentage of males than the survival group. Inflammation levels (CRP and WBC) were high, and dehydration was observed.

**TABLE 1:** Patient characteristics

	*n* = 276	Survivors (*n* = 231)	Non‐survivors (*n* = 45)	*p*‐value
**Age, years**	80.8 (12.9)	80.6 (13.6)	81.9 (8.3)	0.548
**Height, m**	1.59 (0.72)	1.54 (0.10)	1.53 (0.11)	0.999
**BMI, kg/m** ^**2**^	17.2 (3.8)	17.3 (3.8)	17.5 (3.9)	0.128
**GNRI**	76.6 (12.8)	78.1 (12.7)	68.6 (10.1)	<0.001
**Male, *n* (%)**	181 (65.6)	144 (62.3)	37 (82.2)	0.010
**Length of hospital stay, stay**	30.6 (26.6)	30.6 (26.1)	30.2 (29.3)	0.919
**Hospitalisation method**				0.109
Walk‐in, *n* (%)	140 (50.7)	122 (52.8)	18 (40.0)	
Emergency transportation, *n* (%)	136 (49.3)	109 (47.2)	27 (60.0)	
**Medical examination subject**				0.805
Respiratory, *n* (%)	122 (44.2)	100 (43.3)	22 (48.9)	
Emergency, *n* (%)	42 (15.2)	37 (16.0)	5 (11.6)	
General examination, *n* (%)	35 (12.7)	30 (13.0)	5 (11.6)	
Others, *n* (%)	77 (27.9)	64 (27.7)	13 (28.9)	
**Aspiration episode**				0.148
None, *n* (%)	167 (60.5)	141 (61.0)	26 (57.8)	
Vomiting, *n* (%)	47 (17.0)	43 (18.6)	4 (8.9)	
Muze, *n* (%)	44 (15.9)	33 (14.3)	11 (24.4)	
Aspiration, *n* (%)	4 (1.4)	2 (0.9)	2 (4.4)	
Gastrostomy, *n* (%)	9 (3.3)	8 (3.5)	1 (2.2)	
Others, *n* (%)	5 (1.9)	4 (1.7)	1 (2.2)	
**Lifestyle before hospitalisation**				0.346
Home, *n* (%)	151 (54.7)	128 (55.4)	23 (51.1)	
Facility (nursing‐care home), *n* (%)	112 (40.6)	94 (40.7)	18 (40.0)	
Hospital, *n* (%)	13 (4.7)	9 (3.9)	4 (8.9)	
**ADL before hospitalisation**				0.431
Freestanding gait, *n* (%)	70 (25.4)	62 (26.8)	8 (17.8)	
Assistance gait, *n* (%)	73 (26.4)	61 (26.4)	12 (26.7)	
Wheelchair, *n* (%)	90 (32.6)	75 (32.5)	15 (33.3)	
Bedridden, *n* (%)	43 (15.6)	33 (14.3)	10 (22.2)	
**Performance status**				0.766
0, *n* (%)	3 (1.1)	3 (1.3)	0 (0.0)	
1, *n* (%)	30 (10.9)	27 (11.7)	3 (6.7)	
2, *n* (%)	57 (20.7)	48 (20.8)	9 (20.0)	
3, *n* (%)	66 (23.9)	55 (23.8)	11 (24.4)	
4, *n* (%)	120 (43.4)	98 (42.4)	22 (48.9)	
**Dementia**				0.389
Normal, *n* (%)	11 (4.0)	9 (3.9)	2 (4.5)	
Boundary, *n* (%)	33 (12.0)	31 (13.4)	2 (4.5)	
Mild, *n* (%)	33 (12.0)	26 (11.3)	7 (15.6)	
Moderate, *n* (%)	91 (33.0)	73 (31.6)	18 (40.0)	
Severe, *n* (%)	108 (39.0)	92 (39.4)	16 (35.6)	
**A‐DROP**				<0.01
Mild, *n* (%)	0 (0.0)	0 (0.0)	0 (0.0)	
Moderate, *n* (%)	151 (54.7)	139 (60.2)	12 (26.7)	
Severe, *n* (%)	103 (37.3)	79 (34.2)	24 (53.3)	
Extremely severe, *n* (%)	22 (8.0)	13 (5.6)	9 (20.0)	
**Hemiplegia, *n* (%)**	53 (19.2)	46 (19.9)	7 (15.6)	0.489
**Hospitalisation within 1 year, *n* (%)**	148 (53.6)	128 (55.4)	20 (44.4)	0.177
**Oxygen therapy, *n* (%)**	189 (68.5)	154 (66.7)	35 (77.8)	0.063
**Laboratory values**				
BUN, mg/dl	26.2 (20.2)	23.9 (15.7)	37.9 (33.2)	<0.001
Total protein, g/dl	6.6 (0.9)	6.7 (0.9)	6.2 (0.8)	<0.001
Serum albumin, g/dl	3.0 (0.6)	3.1 (0.5)	2.5 (0.6)	<0.001
CRP, mg/dl	10.2 (10.7)	9.3 (10.5)	14.3 (10.6)	<0.01
WBC, 10^3^/μl	11.0 (5.2)	11.2 (5.1)	10.4 (5.6)	0.347
**Physical therapy (physiotherapy), *n* (%)**	228 (82.6)	195 (84.4)	33 (73.3)	<0.01
**Initial mobilisation, days**	3.6 (2.9)	3.6 (3.0)	3.3 (2.4)	0.153
**First‐time antibiotic**				0.011
SBT/ABPC, *n* (%)	203 (73.6)	173 (74.9)	30 (66.7)	
CTRX, *n* (%)	33 (12.0)	26 (11.3)	7 (15.6)	
TAZ/PIPC, *n* (%)	20 (7.2)	12 (5.2)	8 (17.7)	
MEPM, *n* (%)	14 (5.1)	14 (6.1)	0 (0.0)	
Others, *n* (%)	6 (2.1)	6 (2.6)	0 (0.0)	
**Outcome**				<0.001
Home, *n* (%)	79 (28.6)	79 (34.2)	0 (0.0)	
Facility (nursing‐care home), *n* (%)	77 (27.9)	77 (33.3)	0 (0.0)	
Hospital, *n* (%)	75 (27.2)	75 (32.5)	0 (0.0)	
Death, *n* (%)	45 (16.3)	0 (0.0)	45 (100.0)	
**ADL at discharge**				<0.001
Freestanding gait, *n* (%)	45 (16.3)	45 (19.5)	0 (0.0)	
Assistance gait, *n* (%)	48 (17.3)	48 (20.8)	0 (0.0)	
Wheelchair, *n* (%)	91 (33.0)	91 (39.4)	0 (0.0)	
Bedridden, *n* (%)	92 (33.4)	47 (20.3)	45 (100.0)	

*Note*: Values were reported as the mean (SD) or number and percentage of subjects.

Abbreviations: ADL, activity of daily living; BMI, body mass index; BUN, blood urea nitrogen; CRP, C‐reactive protein; CTRX, Ceftriaxone; GNRI, Geriatric Nutritional Risk Index; MEPM, Meropenem; SBT/ABPC, Sulbactam/Ampicillin; TAZ/PIPC, Tazobactam/Piperacillin; WBC, white blood cell.

### 3.3 Examination of risk factors for mortality

Univariate logistic regression analysis showed that the GNRI score, sex, A‐DROP score, BUN, total protein, serum albumin and CRP levels were associated with survival or non‐survival (Table 2). Subsequently, all of the above parameters that showed statistical significance in the univariate analysis, plus age, were incorporated into a multivariate logistic regression model for in‐depth analysis. The results showed that the A‐DROP score (odds ratio [OR] = 2.440; 95% confidence interval [CI], 1.400–4.270; *p* < 0.01), GNRI score (OR = 0.383; 95% CI, 0.178–0.824; *p* < 0.05) and sex (OR = 0.365; 95% CI, 0.153–0.869; *p* < 0.05) were the independent early predictors of mortality (Table 3).

**TABLE 2:** Univariate logistic regression analysis with 90‐day mortality as the dependent variable

	Univariate analysis	
	Odds ratio (95% CI)	*p*‐value
**Age**	1.010 (0.982–1.040)	0.547
**Height**	1.880 (0.151–23.3)	0.143
**BMI**	0.935 (0.858–1.020)	0.13
**GNRI**	0.944 (0.919–0.969)	<0.001
**Sex**		
Male	Reference	
Female	0.358 (0.159–0.804)	0.0128
**Hospitalisation method**		
Walk‐in	Reference	
Emergency transportation	1.710 (0.892–3.270)	0.106
**Medical examination subject**		
Respiratory	Reference	
Emergency	0.655 (0.243–1.77)	0.405
General examination	0.806 (0.296–2.200)	0.674
Others	1.200 (0.601–2.410)	0.600
**Aspiration episode**		
None	Reference	
Vomiting	0.427 (0.145–1.250)	0.122
Muze	1.940 (0.896–4.210)	0.092
Aspiration	5.330 (0.730–38.80)	0.099
Gastrostomy	0.634 (0.077–5.190)	0.671
Others	1.290 (0.141–11.80)	0.822
**Lifestyle before hospitalisation**		
Home	Reference	
Facility (nursing‐care home)	0.972 (0.506–1.860)	0.931
Hospital	2.410 (0.708–8.180)	0.16
**ADL before hospitalisation**		
Freestanding gait	Reference	
Assistance gait	1.010 (0.492–2.090)	0.971
Wheelchair	1.040 (0.528–2.050)	0.91
Bedridden	1.710 (0.775–3.790)	0.183
**Performance status**		
0	Reference	
1	0.540 (0.156–1.860)	0.329
2	0.953 (0.430–2.110)	0.906
3	1.040 (0.492–2.180)	0.927
4	1.300 (0.684–2.460)	0.424
**Dementia**		
Normal	Reference	
Boundary	0.300 (0.069–1.300)	0.108
Mild	1.450 (0.588–3.590)	0.418
Moderate	1.440 (0.747–2.790)	0.275
Severe	0.834 (0.429–1.620)	0.592
**A‐DROP**		
Mild	None	
Moderate	Reference	
Severe	2.200 (1.150–4.190)	0.0168
Extremely severe	4.190 (1.670–10.500)	0.00227
**Hemiplegia**		
None	Reference	
Hemiplegia	0.741 (0.311–1.770)	0.498
**Hospitalisation within 1 year**		
None	Reference	
Hospitalisation within 1 year	1.550 (0.817–2.950)	0.179
**Oxygen therapy**		
None	Reference	
Oxygen therapy	1.550 (0.743–3.220)	0.244
**Laboratory values**		
BUN	1.030 (1.010–1.040)	<0.001
Total protein	0.502 (0.337–0.747)	<0.001
Serum albumin	0.157 (0.0799–0.307)	<0.001
CRP	1.040 (1.0100–1.070)	0.011
WBC	1.000 (1.000–1.000)	0.347
**Physical therapy (physiotherapy)**		
Physical therapy	Reference	
None	1.970 (0.930–4.170)	0.076
**Initial mobilisation**	0.953 (0.804–1.130)	0.58
**First‐time antibiotic**		
SBT/ABPC	Reference	
CTRX	1.450 (0.588–3.590)	0.418
TAZ/PIPC	3.950 (1.510–10.30)	0.507
MEPM	<0.000 (0.000–inf)	0.988
Others	<0.000 (0.000–inf)	0.988

*Note*: Values were reported as the mean (SD) or number and percentage of subjects.

Abbreviations: ADL, activity of daily living; BMI, body mass index; BUN, blood urea nitrogen; CI, confidence interval; CRP, C‐reactive protein; CTRX, Ceftriaxone; GNRI, Geriatric Nutritional Risk Index; MEPM, Meropenem; SBT/ABPC, Sulbactam/Ampicillin; TAZ/PIPC, Tazobactam/Piperacillin; WBC, white blood cell.

**TABLE 3:** Multivariate logistic regression analysis with 90‐day mortality as the dependent variable

Model *p* < 0.001, VIF = 1	Odds ratio	SE	*z*	*p* > |*z*|	95% CI
Dependent variable: 90‐day mortality, *n* = 276					
A‐DROP	2.839	0.3979	2.622	0.0087	1.3226–6.3621
GNRI	0.303	0.4889	−2.439	0.0147	0.1061–0.7439
Sex	0.361	0.4367	−2.329	0.0199	0.1443–0.8151

Abbreviations: CI, confidence interval; GNRI, Geriatric Nutritional Risk Index; VIF, variance inflation factor.

### 3.4 Survival curves for 90‐day survival

**FIGURE 2:**
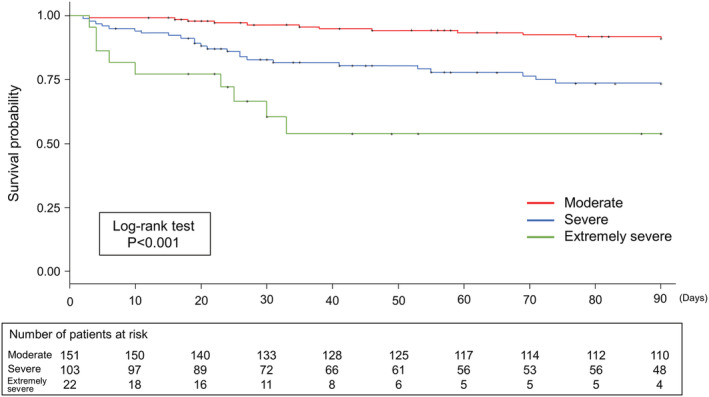
Kaplan–Meier analysis of 90‐day mortality according to the A‐DROP. Classified into three groups according to severity of illness

**FIGURE 3:**
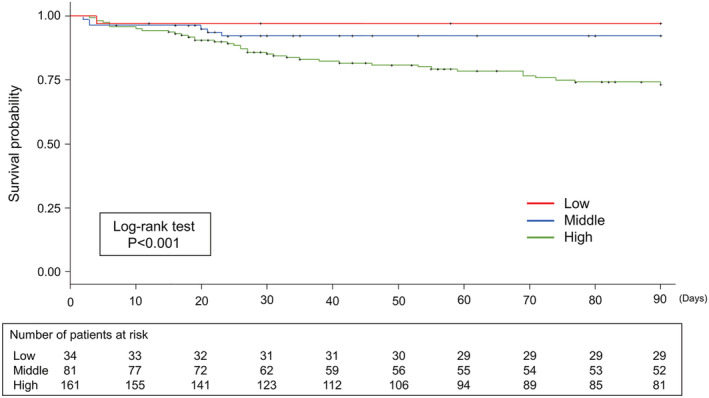
Kaplan–Meier analysis of 90‐day mortality according to the Geriatric Nutritional Risk Index. Classified into three groups according to nutritional risk

Kaplan–Meier analysis was performed for A‐DROP, GNRI and sex. A‐DROP had significantly higher mortality rates with increasing severity of illness (log‐rank test, *p* < 0.001; Figure 2). The GNRI showed significantly higher mortality rates with an increasing risk of nutritional disorders (log‐rank test, *p* < 0.01; Figure 3). Men had a significantly lower survival rate than women (log‐rank test, *p* = 0.012).

## 4 DISCUSSION

In this study, we investigated the factors affecting 90‐day survival in patients hospitalised for aspiration pneumonia. Patients with aspiration pneumonia who had dysphagia or aspiration confirmed by modified water swallow test or VideoEndoscopic examination of swallowing were included. Three factors were extracted by multivariate logistic regression analysis: the A‐DROP score, GNRI score and sex. There are few reports on mortality risk factors for aspiration pneumonia. Maruyama et al. reported malnutrition, high PSI score and initial treatment failure as mortality risks in patients with CAP and HCAP.^13^ Our results were similar to those of Maruyama et al. in terms of malnutrition and GNRI, PSI and A‐DROP scores.

The GNRI is an indicator for predicting complications such as bedsores and infections related to malnutrition and mortality in hospitalised elderly patients.^12^ It can be calculated using only the serum albumin level, current weight and ideal weight. GNRI is significantly correlated with anthropometric and biochemical markers of nutritional status such as grip strength and lymphocytes.^14^, ^15^ The GNRI is not a nutritional indicator; rather, it is an indicator of nutritional disorder‐related complications such as infectious complications, bedsores and death. Moreover, it can predict the risk of mortality, infection and pressure ulcers with more sensitivity than nutritional indicators, such as serum albumin and BMI.^12^

The results of this study revealed that the GNRI is a risk factor for 90‐day survival. Malnutrition in the elderly population is associated with adverse medical consequences, contributing to frailty, sarcopenia, morbidity and mortality.^16^ Low nutrition is one of the factors associated with the development, refractoriness and severity of infectious diseases. Low nutritional status has been reported to cause a decrease in acquired immunity, such as a decrease in helper T cell activity and antibody production,^17^ and low nutrition is a risk factor for infections.^18^ Low BMI and serum albumin levels, which are indicators of low nutrition, have been reported to affect life expectancy in the elderly.^19^, ^20^ In elderly hospitalised patients with low malnutrition, there is a risk of infection, such as sepsis,^21^ and it is also a risk of nosocomial infection.^22^ Furthermore, systemic inflammation caused by aspiration pneumonia may elevate muscle protein catabolism, which depletes muscle mass and leads to further muscle decline and weakness.^23^ This extensive loss of protein may ultimately lead to death. A decrease in skeletal muscle mass in patients with aspiration pneumonia can predict mortality rate for 90 days.^24^ In addition, according to a previous study, the undernourished group had higher hospitalisation costs and complications compared with the non‐undernourished group,^25^ suggesting that the effect of undernutrition is significant.

In patients with aspiration pneumonia, factors related to feeding may influence low nutrition. In the past, Ney et al. reported that dysphagia increased the risk of hyponutrition in the elderly.^26^ However, the association between dysphagia and death from aspiration pneumonia has not yet been reported, and this association needs to be further studied.

This study presented the clinical significance of nutritional risk assessment using the GNRI in patients with aspiration pneumonia. The results showed that low GNRI scores were significantly associated with increased mortality. GNRI score is a useful indicator for stratifying the risk of death in hospitalised patients with aspiration pneumonia. GNRI consists of simple objective measurements such as BMI and serum albumin levels, which can be easily measured on admission for patients with aspiration pneumonia. The GNRI consists of simple objective measurements, BMI and serum albumin, which can be easily obtained on admission in patients with aspiration pneumonia. The GNRI is a significantly useful index for the prognostic prediction of patients with aspiration pneumonia.

Severity of illness was an important risk factor and was associated with increased mortality, similar to the results of other studies.^27^ The A‐DROP score is a modified version of the CURB‐65 score proposed by the Japanese Respiratory Society in 2006.^9^ Although Maruyama et al. used the PSI score as a severity classification, we used the A‐DROP score in this study. The PSI score divides patients into five classes based on a total score of 20 factors, including age, history, abnormalities in physical examination and laboratory findings, and it is the most accurate way to determine the severity of the disease.^28^ However, it is inconvenient because of its large number of items. With the A‐DROP, an immediate decision can be made because it is a five‐item assessment. The predictive power of the A‐DROP as a pneumonia severity classification is similar to that of the PSI.^10^, ^29^ In a study of 1875 patients with CAP, the 30‐day mortality rate increased with increasing severity in A‐DROP (0%, mild; 3.1%, moderate; 9.9%, severe; 19.6%, extremely severe).^29^ The guidelines recommend modifying the treatment according to the severity of the disease. In fact, an association between the pneumonia guideline‐recommended treatment group and lower hospital mortality has been reported.^30^ The results suggest the necessity of treatment selection according to the severity of the A‐DROP.

This study has the following limitations: This was a single‐centre study, and because it was a retrospective study, only data at the time of hospitalisation were collected. To improve the care and rehabilitation of the increasing number of patients with aspiration pneumonia, it is necessary to collect detailed data in a multicentre, prospective study.

Moreover, we investigated the risk factors that affect 90‐day survival in patients with aspiration pneumonia. Three factors were extracted by multivariate logistic regression analysis: the A‐DROP score, GNRI score and sex.

In conclusion, the results of this study suggest that nutritional status and the severity of pneumonia are important factors that predict life expectancy in patients with aspiration pneumonia. While it is natural to assess the severity of the disease at the time of admission, nutritional status should also be assessed to predict the patient's outcome.

## CONFLICT OF INTEREST

The authors declare that they have no known competing financial interests or personal relationships that could have appeared to influence the work reported in this paper.

This study was registered with the University Hospital Medical Information Network Clinical Trial Registry (UMIN 000046923).

## ETHICS STATEMENT

This study was approved by the Ethics Committee of Seirei Mikatahara General Hospital (Approval Number 17‐50). At Seirei Mikatahara General Hospital, we explained the unintended use of medical records at the time of the first visit both verbally and in writing. In this study, we only included patients who provided informed consent for the unintended use of medical records.

## AUTHOR CONTRIBUTIONS

**Yorihide Yanagita**: Conceptualisation; data curation; formal analysis; funding acquisition; investigation; methodology; project administration; resources; software; validation; writing—original draft; writing—review and editing. **Shinichi Arizono**: Conceptualisation; formal analysis; methodology; project administration; supervision; validation; writing—review and editing. **Yuichi Tawara**: Supervision; writing—review and editing. **Masaki Oomagari**: Data curation; formal analysis; investigation; writing—review and editing. **Hikaru Machiguchi**: Data curation; formal analysis; investigation; writing—review and editing. **Koshi Yokomura**: Data curation; resources; supervision; writing—review and editing. **Norimasa Katagiri**: Supervision; writing—review and editing. **Yuki Iida**: Conceptualisation; formal analysis; funding acquisition; methodology; project administration; supervision; validation; writing—review and editing.

## Data Availability

The data that support the finding of this study are available from the corresponding author upon reasonable request.

## References

[1] Marik PE . Aspiration pneumonitis and aspiration pneumonia. N Engl J Med. 2001;344(9):665‐671. 11228282 10.1056/NEJM200103013440908

[2] Teramoto S , Fukuchi Y , Sasaki H , Sato K , Sekizawa K , Matsuse T . High incidence of aspiration pneumonia in community‐ and hospital‐acquired pneumonia in hospitalized patients: a multicenter, prospective study in Japan. J Am Geriatr Soc. 2008;56(3):577‐579. doi:10.1111/j.1532-5415.2008.01597.x 18315680

[3] Komiya K , Rubin BK , Kadota JI , et al. Prognostic implications of aspiration pneumonia in patients with community acquired pneumonia: a systematic review with meta‐analysis. Sci Rep. 2016;6(1):38097. doi:10.1038/srep38097 27924871 PMC5141412

[4] Cameron JL , Mitchell WH , Zuidema GD . Aspiration pneumonia. Clinical outcome following documented aspiration. Arch Surg. 1973;106(1):49‐52. doi:10.1001/archsurg.1973.01350130051011 4565234

[5] Langmore SE , Skarupski KA , Park PS , Fries BE . Predictors of aspiration pneumonia in nursing home residents. Dysphagia. 2002;17(4):298‐307. doi:10.1007/s00455-002-0072-5 12355145

[6] Kohno S , Imamura Y , Shindo Y , et al. Clinical practice guidelines for nursing‐ and healthcare‐associated pneumonia (NHCAP) [Complete Translation]. Respir Investig. 2013;51(2):103‐126. doi:10.1016/j.resinv.2012.11.001 23790739

[7] Oken MM , Creech RH , Tormey DC , et al. Toxicity and response criteria of the eastern cooperative oncology group. Am J Clin Oncol. 1982;5(6):649‐655. doi:10.1097/00000421-198212000-00014 7165009

[8] Nishimura T , Kobayashi T , Hariguchi S , et al. Scales for mental state and daily living activities for the elderly: clinical behavioral scales for assessing demented patients. Int Psychogeriatr. 1993;5(2):117‐134. doi:10.1017/S1041610293001462 8292766

[9] Miyashita N , Matsushima T , Oka M , Japanese Respiratory S . The JRS guidelines for the management of community‐acquired pneumonia in adults: an update and new recommendations. Intern Med. 2006;45(7):419‐428. doi:10.2169/internalmedicine.45.1691 16679695

[10] Usui K , Tanaka Y , Noda H , Ishihara T . Comparison of three prediction rules for prognosis in community acquired pneumonia: Pneumonia Severity Index (PSI), CURB‐65, and A‐DROP. Nihon Kokyuki Gakkai Zasshi. 2009;47(9):781‐785. 19827581

[11] Shindo Y , Sato S , Maruyama E , et al. Comparison of severity scoring systems A‐DROP and CURB‐65 for community‐acquired pneumonia. Respirology. 2008;13(5):731‐735. doi:10.1111/j.1440-1843.2008.01329.x 18713094

[12] Bouillanne O , Morineau G , Dupont C , et al. Geriatric nutritional risk index: a new index for evaluating at‐risk elderly medical patients. Am J Clin Nutr. 2005;82(4):777‐783. doi:10.1093/ajcn/82.4.777 16210706

[13] Maruyama T , Fujisawa T , Okuno M , et al. A new strategy for healthcare‐associated pneumonia: a 2‐year prospective multicenter cohort study using risk factors for multidrug‐resistant pathogens to select initial empiric therapy. Clin Infect Dis. 2013;57(10):1373‐1383. doi:10.1093/cid/cit571 23999080

[14] Poulia KA , Yannakoulia M , Karageorgou D , et al. Evaluation of the efficacy of six nutritional screening tools to predict malnutrition in the elderly. Clin Nutr. 2012;31(3):378‐385. doi:10.1016/j.clnu.2011.11.017 22182948

[15] Cereda E , Vanotti A . The new geriatric nutritional risk index is a good predictor of muscle dysfunction in institutionalized older patients. Clin Nutr. 2007;26(1):78‐83. doi:10.1016/j.clnu.2006.09.007 17067726

[16] Sánchez‐Rodríguez D , Marco E , Annweiler C , et al. Malnutrition in postacute geriatric care: basic ESPEN diagnosis and etiology based diagnoses analyzed by length of stay, in‐hospital mortality, and functional rehabilitation indexes. Arch Gerontol Geriatr. 2017;73:169‐176. doi:10.1016/j.archger.2017.07.010 28822255

[17] Chandra RK . Numerical and functional deficiency in T helper cells in protein energy malnutrition. Clin Exp Immunol. 1983;51(1):126‐132. 6219837 PMC1536761

[18] Scrimshaw NS . Effect of infection on nutrient requirements. Am J Clin Nutr. 1977;30(9):1536‐1544. doi:10.1093/ajcn/30.9.1536 331935

[19] Potter JF , Schafer DF , Bohi RL . In‐hospital mortality as a function of body mass index: an age‐dependent variable. J Gerontol. 1988;43(3):M59‐M63. doi:10.1093/geronj/43.3.M59 3361089

[20] Tsugane S , Sasaki S , Tsubono Y . Under‐ and overweight impact on mortality among middle‐aged Japanese men and women: a 10‐y follow‐up of JPHC study cohort I. Int J Obes Relat Metab Disord. 2002;26(4):529‐537. doi:10.1038/sj.ijo.0801961 12075580

[21] Potter J , Klipstein K , Reilly JJ , Roberts M . The nutritional status and clinical course of acute admissions to a geriatric unit. Age Ageing. 1995;24(2):131‐136. doi:10.1093/ageing/24.2.131 7793335

[22] Paillaud E , Herbaud S , Caillet P , Lejonc JL , Campillo B , Bories PN . Relations between undernutrition and nosocomial infections in elderly patients. Age Ageing. 2005;34(6):619‐625. doi:10.1093/ageing/afi197 16267189

[23] Wilmore DW . Catabolic illness. Strategies for enhancing recovery. N Engl J Med. 1991;325(10):695‐702. doi:10.1056/NEJM199109053251005 1908058

[24] Maeda K , Akagi J . Muscle mass loss is a potential predictor of 90‐day mortality in older adults with aspiration pneumonia. J Am Geriatr Soc. 2017;65(1):e18‐e22. doi:10.1111/jgs.14543 27858956

[25] Reilly JJ Jr , Hull SF , Albert N , Waller A , Bringardener S . Economic impact of malnutrition: a model system for hospitalized patients. JPEN J Parenter Enteral Nutr. 1988;12(4):371‐376. doi:10.1177/0148607188012004371 3138447

[26] Ney DM , Weiss JM , Kind AJ , Robbins J . Senescent swallowing: impact, strategies, and interventions. Nutr Clin Pract. 2009;24(3):395‐413. doi:10.1177/0884533609332005 19483069 PMC2832792

[27] El‐Solh AA , Aquilina AT , Dhillon RS , Ramadan F , Nowak P , Davies J . Impact of invasive strategy on management of antimicrobial treatment failure in institutionalized older people with severe pneumonia. Am J Respir Crit Care Med. 2002;166(8):1038‐1043. doi:10.1164/rccm.200202-123OC 12379545

[28] Fine MJ , Auble TE , Yealy DM , et al. A prediction rule to identify low‐risk patients with community‐acquired pneumonia. N Engl J Med. 1997;336(4):243‐250. doi:10.1056/NEJM199701233360402 8995086

[29] Kohno S , Seki M , Watanabe A . Evaluation of an assessment system for the JRS 2005: A‐DROP for the management of CAP in adults. Intern Med. 2011;50(11):1183‐1191. doi:10.2169/internalmedicine.50.4651 21628933

[30] McCabe C , Kirchner C , Zhang H , Daley J , Fisman DN . Guideline‐concordant therapy and reduced mortality and length of stay in adults with community‐acquired pneumonia: playing by the rules. Arch Intern Med. 2009;169(16):1525‐1531. doi:10.1001/archinternmed.2009.259 19752411

